# The *RAD51 *and *DMC1 *homoeologous genes of bread wheat: cloning, molecular characterization and expression analysis

**DOI:** 10.1186/1756-0500-3-245

**Published:** 2010-09-29

**Authors:** Upendra Kumar Devisetty, Katie Mayes, Sean Mayes

**Affiliations:** 1 Department of Plant and Crop sciences, School of Biosciences, Sutton Bonington Campus, University of Nottingham, Loughborough LE12 5RD, UK; 2 Department of Plant Biology, 1 Shields Ave, University of California Davis, CA 95616, USA

## Abstract

**Background:**

Meiotic recombination in eukaryotes requires two homologues of the *E. coli *RecA proteins: Rad51 and Dmc1. Both proteins play important roles in the binding of single stranded DNA, homology search, strand invasion and strand exchange. Meiotic recombination has been well studied in Arabidopsis, rice, maize and the orthologues of *RAD51 *and *DMC1 *have been characterized. However genetic analysis of the *RAD51 *and *DMC1 *genes in bread wheat has been hampered due to the absence of complete sequence information and because of the existence of multiple copies of each gene in the hexaploid wheat genome.

**Findings:**

In this study we have identified that *TaRAD51 *and *TaDMC1 *homoeologues are located on group 7 and group 5 chromosomes of hexaploid wheat, respectively. Comparative sequence analysis of cDNA derived from the *TaRAD51 *and *TaDMC1 *homoeologues revealed limited sequence divergence at both the nucleotide and the amino acid level. Indeed, comparisons between the predicted amino acid sequences of *TaRAD51 *and *TaDMC1 *and those of other eukaryotes reveal a high degree of evolutionary conservation. Despite the high degree of sequence conservation at the nucleotide level, genome-specific primers for cDNAs of *TaRAD51 *and *TaDMC1 *were developed to evaluate expression patterns of individual homoeologues during meiosis. QRT-PCR analysis showed that expression of the *TaRAD51 *and *TaDMC1 *cDNA homoeologues was largely restricted to meiotic tissue, with elevated levels observed during the stages of prophase I when meiotic recombination occurs. All three homoeologues of both strand-exchange proteins (*TaRAD51 *and *TaDMC1*) are expressed in wheat.

**Conclusions:**

Bread wheat contains three expressed copies of each of the *TaRAD51 *and *TaDMC1 *homoeologues. While differences were detected between the three cDNA homoeologues of *TaRAD51 *as well as the three homoeologues of *TaDMC1*, it is unlikely that the predicted amino acid substitutions would have an effect on the protein structure, based on our three-dimensional structure prediction analyses. There are differences in the levels of expression of the three homoeologues of *TaRAD51 *and *TaDMC1 *as determined by QRT-PCR and if these differences are reflected at the protein level, bread wheat may be more dependent upon a particular homoeologue to achieve full fertility than all three equally.

## Background

Meiosis is central to all sexually reproducing living organisms and is a highly conserved process. During meiosis a single round of DNA replication is followed by two rounds of cell division resulting in the formation of four daughter cells each having half the chromosome number of the parent. Recombination at meiosis is pivotal not only for creating genetic diversity through crossing-over but also has a mechanistic role ensuring physical connections in the form of chiasmata that allow the chromosomes to segregate accurately during the first division of meiosis. Bacterial RecA was the first gene to be cloned from *E. coli *that was shown to play an important role in recombination [1,2]. Meiotic recombination in eukaryotic cells generally requires two homologues of the *E. coli *RecA protein - Rad51 and Dmc1 - that were first identified in *Saccharomyces cerevisiae *[3,4]. The Rad51 protein forms a nucleofilament with ATP-dependant strand-exchange activity, which has been shown to have roles in meiosis, homologous recombination and DSB repair [5]. Dmc1 also forms a nucleofilament in cooperation with Rad51 and is involved in generating hybrid joint molecules specifically in meiosis [3]. The *RAD51 *gene exists as a single copy in tomato and Arabidopsis, while maize and *Physcomitrella patens *have two copies each [6-11]. The *DMC1 *gene sequences have been reported only in a few plant species. While the Arabidopsis genome contains one copy of the *DMC1 *gene [12], rice has two copies, *OsDMC1A *and *OsDMC1B*, which are located on chromosomes 12 and 11, respectively, most likely produced by chromosomal duplication [13].

Common wheat or bread wheat is an allohexaploid composed of three genomes, each consisting of seven chromosomes (AABBDD; 2*n *= 6*x *= 42). Information on the genes and gene homoeologues of *RAD51 *and *DMC1 *in bread wheat is limited, primarily due to the added complexity of polyploidy. Given that *RAD51 *and *DMC1 *gene orthologues are reported to be involved in meiotic recombination in other cereal species and also the model plant *Arabidopsis thaliana*, we have investigated their orthologues in bread wheat. A previous study on meiotic recombination genes in wheat reported the existence of only one copy of *TaDMC1 *gene [GenBank accession number EU915561] and two copies of *TaRAD51 *gene - *TaRAD51A1 *[GenBank accession number EU915557] and *TaRAD51A2 *[GenBank accession number EU915558] [14]. We report here for the first time the existence of three homoeologous copies of the *TaRAD51 *and *TaDMC1 *genes, their genomic location and expression patterns in inflorescence and vegetative tissues in bread wheat. These observations in the Chinese Spring wheat do not completely agree with the results reported earlier [14] and these apparent differences are discussed in this paper.

## Methods

### Plant materials and DNA isolation

Wild type hexaploid wheat (*Triticum aestivum *c.v Highbury) and Nullisomic-Tetrasomic (NT) wheat stocks (*Triticum aestivum *c.v Chinese Spring) were kindly provided by Prof John Snape and Dr Steve Reader at the John Innes Centre (Norwich, UK). The NT stocks, originally developed by Sears [15], were used for chromosomal assignment of the putative homoeologous genes. Lines Nullisomic for genome A chromosomes were tetrasomics either for B or D chromosomes, lines Nullisomic for genome B chromosomes were tetrasomics either for A or D chromosomes and Nullisomic for genome D chromosomes were tetrasomics either for A or B chromosomes.

Seeds of wheat were first germinated on moistened filter papers in Petri dishes incubated at 20°C in a growth chamber. When coleoptile length had reached ~1 cm, seedlings were transplanted to 13 cm pots containing John Innes No. 3 compost and grown under standard glasshouse conditions. Total genomic DNA was extracted from young leaf tissue (2 - 4 weeks post emergence) using the GenElute™ Plant Genomic DNA Miniprep kit (Sigma Aldrich) according to the manufacturer's instructions.

### RNA extraction and first strand cDNA synthesis

Total RNA was isolated from the following tissues: root tips (RT, 14 days after germination), mature leaf (L) and immature inflorescence material (I). In addition, meiotically staged anthers were collected for RNA extraction from anther (microspore) tissues. Stages included are: pre-meiosis (PM), leptotene to pachytene (LP), diplotene to anaphase I (DA), telophase I to telophase II (TT), tetrads (T) and immature pollen (IP). Extractions used Trizol reagent (Gibco-BRL) according to the manufacturer's instructions. The isolated RNA was treated with RQ1 RNase-Free DNase (Promega) to remove any residual genomic DNA according to the manufacturers' instructions and first strand cDNA was synthesized using Superscript III Reverse Transcriptase (Invitrogen) using standard methods.

### Isolation of full-length cDNA sequences of wheat *RAD51 *and *DMC1 *genes

Wheat *RAD51 *and *DMC1 *genes had not been reported at the start of this research, so in order to isolate the wheat *RAD51 *cDNA sequence, the rice *OsRAD51A1 *cDNA sequence [Gen Bank accession number ABO8O261.2] was retrieved from the GenBank database (http://www.ncbi.nlm.nih.gov/genbank) and used to design a total of four primer sets to amplify different sized fragments covering the entire rice cDNA sequence, including 3' and 5' UTR sequences (Table 1). These were then used in RT-PCR using wheat immature inflorescence cDNA as the template. The RT-PCR products were cloned, sequenced and aligned into contigs to obtain the full-length *TaRAD51 *cDNA. To isolate the full-length wheat *DMC1 *cDNA sequences, primers were designed to the partial *DMC1 *cDNA sequence of wheat [GenBank accession number DQ247845] and 3' and 5' RACE was performed. To generate *TaDMC1 *3' ends, mRNA was reverse transcribed using Oligo(dT_18_) primer (Table 1). After first strand cDNA synthesis, the 3' region of the *TaDMC1 *cDNA was amplified with DGSP1 and the 3' *Not*I anchor primer (Table 1) in the first PCR, and then a nested PCR was done with DGSP2 in combination with the *Not*I anchor primer (Table 1). 5'-RACE to generate *TaDMC1 *5' ends was carried out using a 5'RACE System for Rapid Amplification of cDNA Ends kit (Invitrogen) following the manufacturer's instructions. The PCR products were obtained with AAP or AUAP primers (provided in the kit) and gene specific primers (Table 1). The RT-PCR products were cloned, sequenced and aligned into contigs to obtain the full-length *TaDMC1 *cDNA.

**Table 1:** List of primers used in the present study

Primer name	Primer sequence (5'-3')	Tm°C	Size (bp)	Use
*OsRAD51*Exon 1-2-F	CCTCACATCCCGAGCATCTC	60	171	Cloning of full-length cDNA of wheat *RAD51*
*OsRAD51*Exon 1-2-R	TCACATCAACTGCAGCTATTCCAG			
*OsRAD51 *Exon 8-9-F	GGTAGTGGCCCAAGTGGATG	60	564	
*OsRAD51 *Exon 8-9-R	TGGTGGTCCAATATCACATAGGAG			
*OsRAD51 *Exon 1-7-F	ATGTCGTCGGCGGCGG	65	889	
*OsRAD51 *Exon 1-7-R	CTCATCCGCTAATTTCTGAAGGC			
*OsRAD51 *Exon 2-9-F	AAGTCGATAAGATAAT TGAAGCA	60	879	
*OsRAD51 *Exon 2-9-R	CTGAAACCTTGCTTCAGCT			
NotI-(dT)_18_	AACTGGAAGAATTCGCGGCCGCAGGAA(T)_18_			Cloning of full-length cDNA of wheat *DMC1*
*Not*I(dT) anchor	AACTGGAAGAATTCGCGG			
*TaDMC1 *-DGSP1	GAAGAGCCTTTCAGGCTTCTG	55	700	
*TaDMC1-*DGSP2	GGTGAACTTGCAGAGCGCCAG	55	772	
*TaDMC1-*DGSP3	CTGGCGCTCTGCAAGTTCACC	55	772	
*TaDMC1-*DGSP4	GGAAGACCCAGTTGGCTCAT	55	461	
*TaDMC1-*DGSP5	GGTGAACTTGCAGAGCGTCAG	55	207	
*TaRAD51 *Exon 8-F	CCATGATGGTGGAGACAAGG	65	2500	Amplification of intronic regions in *TaRAD51 *&*TaDMC1 *genes
*TaRAD51 *3'UTR-R	TGGTGGTCCAATATCACATAGGAG			
*TaDMC1*-5'UTR-F	ATGATCCACATTCCACCCGC	65	2500	
*TaDMC1*-Exon 6-R	ATGAGCCAACTGGGTCTTCC			
*iTaRAD51-7A*&*7D*-F	CGGAAGGATTGGTA AAAAAT	55	450 & 500	*TaRAD51 *&*TaDMC1 *genome-specific primers based on intronic regions for localization on wheat genome
*iTaRAD51-7A*&*7D*-R	CACTCAGAAATGACG AAAAAGG			
i*TaRAD51-7B*-F	ATGGTGGAGACAA GGTGAG	63	500	
*iTaRAD51-7B*-R	GTACACTAGCATTAC GGTACAG			
*iTaDMC1-5A*-F	AGCCTCCGCCCCACTTCCTTC	63	500	
*iTaDMC1-5A*-R	ACAAACGCAACACGA GCACACG			
*iTaDMC1-5B*-F	AGCCTTGGCCCCACTTCCTC	63	500	
*iTaDMC1-5B*-R	ACGCGCTGCACGCA CCAAA			
*iTaDMC1-5D*-F	CTCCTCTGACGCAG GCGGA	63	389	
*iTaDMC1-5D*-R	ACGCGCTGCACGC ACCAAA			
*TaRAD51*-UTR-F	CCTCACATCCCGAGCATCTC	63	1528	*TaRAD51 *cDNA full-length primers including UTR's
*TaRAD51*-UTR-R	CTCCTATGTGATATTGGACCACCA			
*eTaRAD51-7A*-F	GGGGATACCTCGTGTATCAGACT	58	328	*TaRAD51 *genome-specific primers based on exons for QRT-PCR analyses
e*TaRAD51*-R	TGGTGGTCCAATATCACATAGGAG			
e*TaRAD51-7B*-F	GATACATCGTGTATCGGACA	58	328	
e*TaRAD51-*R	TGGTGGTCCAATATCACATAGGAG			
*eTaRAD51-7D*-F	TGGGGATACATCGTGTATTGGCCT	65	327	
e*TaRAD51-*R	TGGTGGTCCAATATCACATAGGAG			
*TaDMC1*-UTR-F	ATGATCCACATTCCACCCGC	63	1268	*TaDMC1 *cDNA full-length primers including UTR's
*TaDMC1*-UTR-R	CACTGCAGAAAAGAAATTGGGCAAC			
*eTaDMC1-5A*-F	AGCCTCCGCCCCACTTCCTTC	65	150	*TaDMC1 *genome-specific primers based on exons QRT-PCR analyses
e*TaDMC1*-R	CTTGTCGATGGACTCGAAGCACTC			
*eTaDMC1-5B*-F	AGCCTTGGCCCCACTTC CTC	65	150	
e*TaDMC1*-R	CTTGTCGATGGACTCGAAGCACTC			
*eTaDMC1-5D*-F	TTCTCCTCCAGCAGCACGCGAA	65	150	
e*TaDMC1*-R	CTTGTCGATGGACTCGAAGCACTC			
TUB-F	TCTTCATGGTGGGCTTCGC	55	475	QRT-PCR control primers
TUB-R	CGCCTCGGGTGAACTCCATCT			

### Generation of Genome-specific primers for *TaRAD51 *and *TaDMC1 *using intron sequences

*OsRAD51A1 *[GenBank accession number ABO8O261] and *OsDMC1A *[GenBank accession number AB079873] cDNA sequences were used to predict the exon/intron boundaries of *TaRAD51 *and *TaDMC1*, respectively. Once the boundaries were identified, exon-anchored primers that were expected to amplify products spanning two or three introns were designed (Table 1). Multiple alignment of sequences obtained from the *TaRAD51 *and *TaDMC1 *cDNA exon-anchored primers on Chinese Spring genomic DNA identified three distinct groups of sequences for each gene with respect to the intronic sequences in bread wheat (data not shown). Putative primers that were expected to amplify a particular homoeologue were designed based on the sequence polymorphisms detected and were coupled with a conserved exon-anchored primer in a PCR reaction containing genomic DNA from Chinese Spring and the appropriate nulli-tetrasomic stocks as templates.

### Generation of Genome-specific primers for *TaRAD51 *and *TaDMC1 *sequences at the exon level

Highbury spikelet cDNA was amplified using primers that generate full-length copies of the *TaRAD51 *and *TaDMC1 *genes. A major amplification band, approximately 1.5 kb was obtained with both sets of primers. In the absence of differentially sized fragments, genome-specific primers for *TaRAD51 *and *TaDMC1 *cDNA homoeologues were designed based on sequence polymorphisms identified at the 3' UTRs for *TaRAD51 *cDNA homoeologues and at the 5' UTRs for the *TaDMC1 *cDNA homoeologues and were paired with a conserved gene primer (Table 1). The genome specificity of the primers was tested using appropriate nulli-tetrasomic stocks as templates.

### Comparative amino acid sequence analysis and 3D protein modelling

Comparative amino acid sequence analysis was undertaken using the ClustalW tool of European Bioinformatics Institute (http://www.ebi.ac.uk/Tools/clustalw2). The amino acid sequences were processed using the Swiss model workspace (version 8.05, 2009) [16]. The computed outputs were then used to predict the 3D protein structures using Swiss-Pdb Viewer (DeepView; Version 4.0, 2009) [17].

### SIFT (Sorting Intolerant From Tolerant) analysis

The potential impact of amino-acid changes on the final protein structure were assessed with the SIFT program [18]. SIFT is a program which predicts whether an amino acid substitution in the protein affects its protein function or not. SIFT assumes that important amino acids will be conserved in the protein family and so changes at well-conserved positions tend to be predicted as deleterious. SIFT score ranges from 0 to 1. The amino acid substitution is predicted deleterious if the score is ≤ 0.05 and tolerated if the score is > 0.05. This approach can only give an indication of potential changes to function through mutation.

### Phylogenetic analysis

The alignment of nucleotide sequences and subsequent selection of best-fit molecular evolution model was performed using Molecular Evolutionary Genetics Analysis (MEGA) software (version 5.0) [19]. The construction of a phylogenetic tree was performed under maximum likelihood (ML) using RAxML version 7.2.6 [20] on the CIPRES portal [21] with the following parameters: nucleotide search using the GTR + G model, random seed for initial parsimony inference, rapid bootstrapping with automatic bootstopping function (frequency criterion) followed by the final ML search.

### Quantitative Real-Time PCR

Harvesting and staging of meiotic anthers for QRT-PCR was performed from immature wheat spikes [22]. Harvested material was immediately frozen and stored at -80°C. Eight independent QRT-PCR amplifications were made for the following tissues: PM, LP, DA, TT, T, IP, L and RT. QRT-PCR was performed in triplicates using wheat Tubulin (Table 1) as an internal standard in a total volume of 12 μl reaction that contained 6 μl of Brilliant^® ^SYBR^® ^Green (Qiagen), 50 ng cDNA and 1 μl of an equal mix of forward and reverse gene specific primers (Table 1). The reaction conditions for QRT-PCR were as follows: 15 min at 95°C followed by 45 cycles of 30 s at 95°C, 30 s at gene specific annealing temperature and 30 s at 72°C. Primer sequences and annealing temperatures are given in Table 1.

Q-PCR amplification reactions were performed in the LightCycler^® ^480 instrument (Roche Applied Science, UK) using Brilliant^® ^SYBR^® ^Green (Qiagen) according to the manufacturer's instructions.

### Accession numbers

The cDNA sequences of the *TaRAD51 *and *TaDMC1 *homoeologues were deposited in GenBank at NCBI [GenBank accession numbers FJ594479, FJ594480 and FJ594481 for *Ta*cRAD51-7A, *Ta*cRAD51-7B and *Ta*cRAD51-7D homoeologues, respectively and FJ594476, FJ594477 and FJ594478 for *Ta*cDMC1-5A, *Ta*cDMC1-5B and *Ta*cDMC1-5D homoeologues, respectively)].

## Results and Discussion

### Isolation and genome localization of *RAD51 *and *DMC1 *gene homoeologous sequences of hexaploid wheat

**Figure 1:**
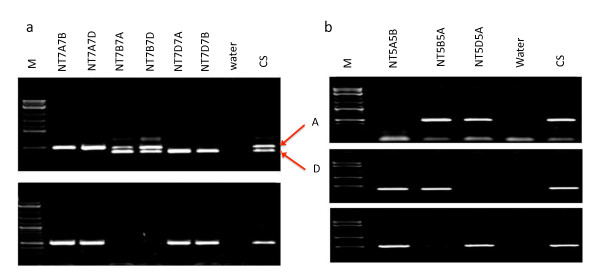
**PCR assay for the chromosomal localization of the *TaRAD51 *and *TaDMC1 *homoeologous loci, respectively, on group 7 and group 5 of hexaploid wheat**. Each primer set designed for *TaRAD51 *was used to amplify NT7A7B, NT7A7D, NT7B7A, NT7B7D, NT7D7A, NT7D7B, water control and CS. (a) *TaRAD51 *(A) and (D) genome-specific primer PCR amplifications. The two bands (indicated by red arrows) in Nullisomic 7B and tetrasomic for 7A and 7D lanes can be allocated either to A or D genomes based on PCR amplification on other Nullisomics (Nullisomics 7A and 7D); *TaRAD51*(B) genome-specific primer PCR amplification and absence of bands in the line nullisomic for 7B and tetrasomic for both 7A and 7D allocates this primer to genome 7B. Each primer set for *TaDMC1 *was used to amplify NT5A5B, NT5B5A, NT5D5A, water control and CS (b) *TaDMC1*(A), (B) & (D) genome-specific primer PCR amplification. Absence of bands in the group 5 Nullisomics allocates the primer to the respective genomes; M = 2-log ladder (NEB). Only group 5 and group 7 of Nulli-tetrasomics are shown.

**Figure 2:**
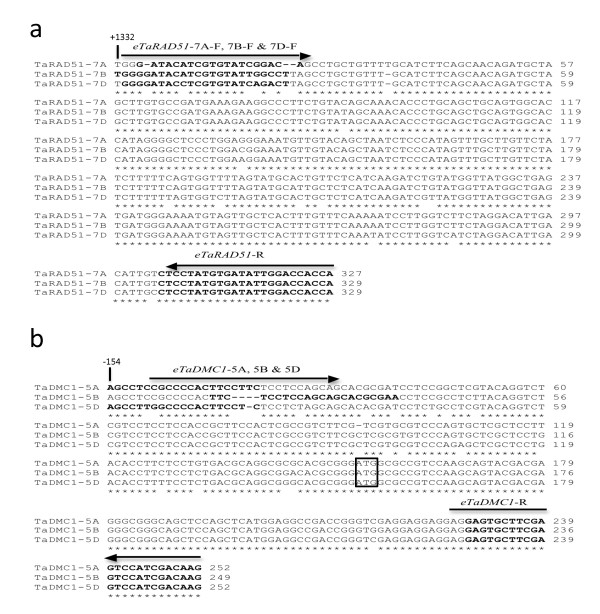
**Alignment of 3'- and 5'-UTR sequences of *TaRAD51 *and *TaDMC1 *homoeologues from hexaploid wheat indicating the location of genome specific primers**. (a) *TaRAD51 *cDNA genome-specific primer design. (b) *TaDMC1 *cDNA genome-specific primer design. Deletions are shown by *dashes*. The positions of the 3' and 5' UTRs in relation to the start codon 'A' are indicated at the top of the sequence. The sequence of the forward primer and complementary sequences of the reverse primers are shown in bold. The black arrows indicate the position of forward and reverse primers.

**Figure 3:**
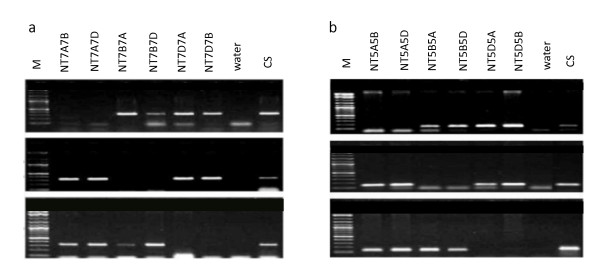
**PCR assay for genome specificity of *TaRAD51 *and *TaDMC1 *cDNA homoeologues in bread wheat**. Each primer set for *TaRAD51 *was used to amplify NT7A7B, NT7A7D, NT7B7A, NT7B7D, NT7D7A, NT7D7B, water control and CS (a) *TaRAD51*(A), (B) & (D) genome-specific primer PCR amplification. Each primer set for *TaDMC1 *was used to amplify NT5A5B, NT5A5D, NT5B5A, NT5B5D, NT5D5A, NT5D5B, water control and CS.(b) *TaDMC1*(A), (B) & (D) genome-specific primer PCR amplification. Absence of bands in the group 7 & 5 Nullisomics allocates the primer to the respective genomes; M = 2-log ladder (NEB).

'Highbury' wheat spikelet cDNA amplifications resulted in the isolation of the full-length *RAD51 *and *DMC1 *cDNA sequences from hexaploid wheat (see methods). In order to localize the isolated *RAD51 *and *DMC1 *cDNA sequences on hexaploid wheat, genome specific primers were developed in intronic regions and the genome specificity was confirmed using nulli-tetrasomic analysis. The analysis revealed that the *TaRAD51 *and *TaDMC1 *sequences were located on group 7 and group 5 chromosomes, respectively, with a copy in each of the A, B and D genomes (Figure 1). Exon specific genome specific primers were developed based on sequence polymorphisms identified (Figure 2) and the genome specificity was confirmed using group 7 and group 5 nulli-tetrasomics of wheat (Figure 3). Later full-length cDNA sequences of the *TaRAD51 *and *TaDMC1 *homoeologues were isolated using these primers and the sequences were submitted to NCBI database. The locations of *TaRAD51 *homoeologues cloned here contrast with a previous report [14] in bread wheat that *TaRAD51 *exists as two paralogues - *TaRAD51A1 *and *TaRAD51A2 *on group 7.

### *TaRAD51 *and *TaDMC1 *cDNA homoeologues are highly conserved at the amino acid level

**Figure 4:**
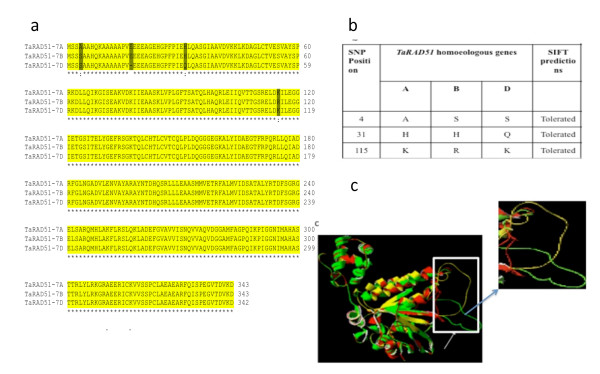
**The deduced amino acids alignments of *TaRAD51 *homoeologous proteins and their 3D models**. (a) Multiple alignments of the three *TaRAD51 *sequences identified in the three bread wheat genomes (A, B and D). Conserved amino acids are indicated by black with a yellow background. The amino acid differences between the three cDNA homoeologous proteins are indicated by black with a grey background. (b) SIFT predictions for the amino acid substitutions for the three cDNA homoeologues of *TaRAD51*. (c) The 3D structure of TaRad51-7A is represented in red, TaRad51-7B in green and TaRad51-7D in yellow. Blue arrow shows the magnified image of the side chains of three TaRad51 homoeologue proteins, which indicates the only structural dissimilarity.

Sequence alignment of the *TaRAD51 *cDNA homoeologues at nucleotide and amino acid level revealed 97% and 99% identity respectively. Two of the *TaRAD51 *cDNA homoeologues (*TaRAD51-7A *and -*7B*) have a continuous ORF of 1032 bp, capable of encoding a protein of 343 amino acids and the *TaRAD51-7D *ORF has a deletion of three nucleotides at the N-terminal end, thus putatively encoding a predicted protein of 342 amino acids residues. Further analysis of the three translated *TaRAD51 *cDNA homoeologues revealed that this leads to the expected deletion of one amino acid at position 17 corresponding to an 'E' residue (Glutamic acid) and single amino acid substitutions are also expected between the three homoeologues at positions 4 (A, compared with B&D), 31 (A&B, compared with D) and 115 (A&D, compared with B) (Figure 4a). SIFT predictions suggested that the amino acid substitutions are not expected to affect protein 3D structure for the three cDNA homoeologues of *TaRAD51 *(Figure 4b). We have also found that the previously reported [14]*TaRAD51A1 *is identical to the *TaRAD51-7D *homoeologue isolated here and that the reported *TaRAD51A2 *sequence appears to be a truncated version of the *TaRAD51-7A *homoeologue albeit with a few amino acid differences at the truncated end (listed in Additional file 1). However the paralogous nature of the reported *TaRAD51A1/A2 *[14] was not observed in this study. This supports the idea that there is only one copy of *TaRAD51 *per haploid wheat genome, at least for the wheat genotype studied here. The predicted 3D homoeologue overlays superimposed onto each other TaRad51 revealed there is a high level of predicted conservation for secondary and tertiary structures (Figure 4c). The only noticeable structural dissimilarity observed between the three cDNA homoeologues is in peptide loops seen between α-helix 13 and α-helix 14 (indicated by the white arrow in Figure 4c)

**Figure 5:**
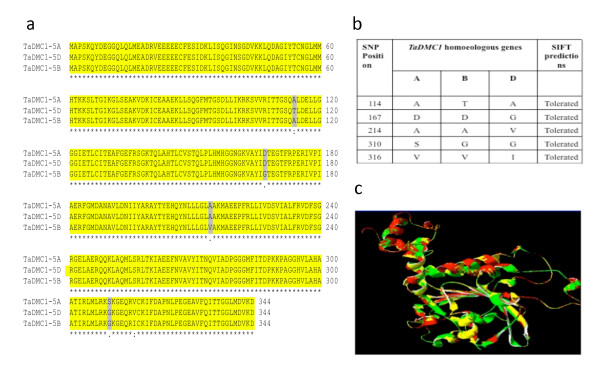
**The deduced amino acids alignments of *TaDMC1 *homoeologous proteins and their 3D models**. (a) Multiple alignments of the three *TaDMC1 *sequences identified in the three bread wheat genomes (A, B and D). Conserved amino acids are indicated by black with a yellow background. The amino acid differences between the three homoeologous proteins are indicated by black with a grey background. (B) SIFT predictions for the amino acid substitutions for the three cDNA homoeologues of *TaDMC1*. (C) The predicted 3D structure of TaDmc1-7A is represented in red, TaDmc1-7B in green and TaDmc1-7D in yellow.

Conceptual translation and subsequent sequence alignment of *TaDMC1 *showed 97% nucleotide sequence identity and 98% identity at the amino acid level between the three translated homoeologous copies. All cDNA homoeologues of the *TaDMC1 *have a continuous ORF of 1035 bp which encodes a predicted protein of 344 amino acids. Further analysis of the three translated *TaDMC1 *cDNA homoeologues revealed that the only differences identified between the three cDNA homoeologues were due to nucleotide differences predicted to produce single amino acid substitutions at positions 114 (A&D, compared with B), 166 (A&B compared with D), 214 (A&B compared with D), 310 (A, compared with B&D) and 316 (A&B, compared with D); no insertions or deletions were detected (Figure 5a). SIFT predictions for the three translated homoeologues of *TaDMC1 *suggested that the amino acid substitutions are not expected to affect protein secondary and tertiary structure (Figure 5b). Multiple alignment of the amino acid sequence of the three translated homoeologues of *TaDMC1 *with the previously reported *TaDMC1 *cDNA sequence [14] revealed that the reported *TaDMC1 *is the D genome homoeologue (data not shown). The predicted 3D overlays for the translated *TaDMC1 *homoeologues superimposed onto each other revealed very high levels of conservation for secondary and tertiary structures (Figure 5c).

### Phylogenetic analysis of *TaRAD51 *and *TaDMC1 *cDNA homoeologues reveals evolutionary conservation

**Figure 6:**
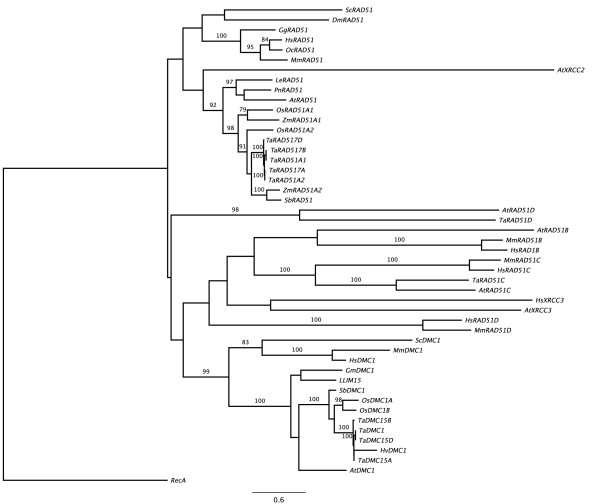
**Evolutionary tree of Rad51 and Dmc1 proteins**. Phylogenetic tree obtained from a nucleotide alignment of cDNAs derived from the *TaRAD51 *and *TaDMC1 *homoeologues together with a wide range of *RAD51 *and *DMC1 *orthologues using the Maximum Likelihood method.

To further analyze the level of conservation and evolutionary relationships of the Rad51 and Dmc1 predicted proteins across a diverse range of species and to examine the predicted identity of the *TaRAD51 *and *TaDMC1 *cDNA homoeologues, previously annotated database entries (listed in Additional file 2) were used to construct a phylogenetic tree. The tree identified three main branches, with Rad51 and Dmc1 members clustered into two separate branches with Rad51B, Rad51C, Rad51D, Xrcc2 and Xrcc3 clustered into a third (Figure 6). This is consistent with the reports that Rad51 and Dmc1 are paralogues and likely descendants of the ancestral RecA gene [23]. As expected, the three cDNA homoeologues of *TaRAD51 *and *TaDMC1 *clustered together and fall into their respective branches. The strongest similarity was found between the nucleotide sequences of wheat and rice *RAD51 *and *DMC1*. This provides strong evidence that the cDNA homoeologues isolated in this study are the true orthologues of the rice *RAD51 *and *DMC1 *genes.

This study also found that there was only one copy of *TaRAD51 *per haploid genome of wheat in contrast to two copies of the *RAD51 *gene reported in rice (*OsRAD51A1/A2*; rice chromosomes 11 and 12) and maize (*ZmRAD51A1/A2*; maize chromosomes 7 and 8) [8,13]. The presence of two *RAD51 *copies in rice and maize and only one copy of *RAD51 *per genome in wheat may argue for gene loss in the ancestors of wheat. This conclusion is supported by the phylogenetic tree (Figure 6). The genomes of rice and maize contain both products of a predicted earlier gene duplication before divergence of the two species i.e *RAD51A1 *and *RAD51A2*, while all three of the *RAD51 *genes in wheat appear to be homologues of the *RAD51A2 *gene, suggesting that the *RAD51A1 *lineage has been lost in bread wheat. There are also two versions of the *DMC1 *gene in rice and maize and only one in the haploid wheat genome. This may have a similar origin, but the greater conservation of *DMC1 *in these species makes an assessment more difficult and the presence of two copies may simply reflect a local duplication of R11 and R12 in rice and the fact that maize is an ancient tetraploid. In both case, it is worth noting that *DMC1 *homologues are more highly conserved than the *RAD51 *homologues, arguing for a higher level of selective pressure preventing mutation or a more recent duplication event.

### All homoeologues of *TaRAD51 *and *TaDMC1 *genes were highly expressed during prophase I of meiosis

**Figure 7:**
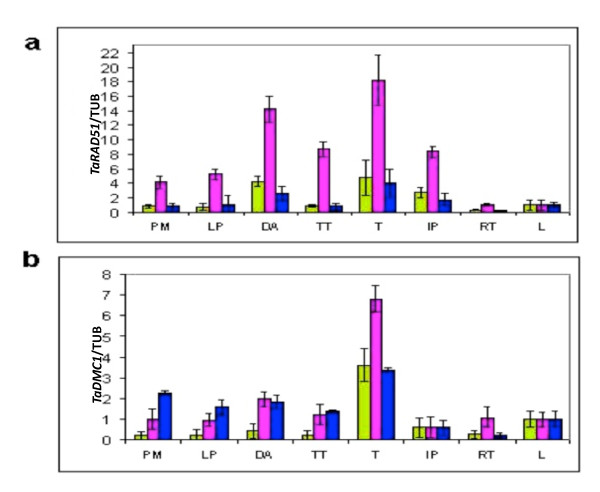
**QRT-PCR expression analysis of the *TaRAD51 *and *TaDMC1 *homoeologous genes**. (a) The green bars represents *TaRAD51(A)*, pink bar represents *TaRAD51(B) *and blue bar represents *TaRAD51(D)*. (B) The green bars represents *TaDMC1(A)*, pink bar represents *TaDMC1(B) *and blue bar represents *TaDMC1(D)*. Abbreviations used: PM, pre-meiotic interphase; LP, leptotene-pachytene, DA, diplotene-anaphase I; TT, telophase I-telophase II; T, tetrad; IP, immature pollen; RT, root tips; L, leaves

The QRT-PCR analysis of *TaRAD51 *cDNA homoeologues indicated that all the three homoeologues were expressed in both vegetative and meiotic tissues (Figure 7a). Comparatively higher expression levels were observed in meiotic stages than vegetative tissues (Figure 7a, stages PM to TT). There is a sharp drop in expression of the *TaRAD51 *cDNA homoeologue transcripts at the end of Meiosis I but then expression increases again at the Tetrad stage. There was however differences in expression level among the three cDNA homoeologues, particularly during meiotic stages. This may suggest that the individual homoeologues contribute to differing extents in meiotic recombination. The expression level of *TaRAD51-7B *was significantly higher than *TaRAD51-7A *and *TaRAD51-7D *in all meiotic stages suggesting it could be the version with the greatest role in meiosis.

QRT-PCR analysis of *TaDMC1 *cDNA homoeologues indicated that all three homoeologues were expressed in both vegetative and meiotic tissues (Figure 7b). However higher expression levels were found in meiotic stages (Figure 7b, stages PM to TT) compared to non-meiotic tissues and we observed an increase in expression of all three *TaDMC1 *cDNA homoeologues during the Tetrad stage. There were differences in the expression levels among all three cDNA homoeologues of *TaDMC1 *in the observed tissues, which suggests that they could contribute to differing extents in meiotic recombination. Both *TaDMC1-5B *and *TaDMC1-5D *homoeologues were highly expressed in all meiotic stages, although not the Tetrad stage, compared to the transcript level of *TaDMC1-5A *suggesting they could be the versions with the greatest role in meiosis.

Overall, we have reported the cloning and characterisation of the hexaploid wheat versions of the major strand-exchange proteins *DMC1 *and *RAD51*. Primer tests have been developed which are specific to individual homoeologues and these have been used to characterise the levels of genome-specific expression in a range of tissues through Q-PCR. The same primers could be used to screen mutation and deletion stocks of wheat to detect loss-of-function mutations, allowing *in planta *characterisation of the effect of these mutations on meiosis.

## Abbreviations

AA: Amino Acids; DSB: Double Strand Breaks; NT: Nulli-Tetrasomics; RACE: Rapid Amplification of cDNA Ends; CS: Chinese Spring; RT-PCR: Reverse Transcription Polymerase Chain Reaction.

## Competing interests

The authors declare that they have no competing interests.

## Authors' contributions

UKD conducted the actual research, analyzed the data pertaining to experiments and drafted the manuscript. KM designed the primers for the isolation of *TaRAD51 *and *TaDMC1 *genes. SM guided the research and proofread this manuscript.

## Supplementary Material

Additional file 1 **Alignment and comparison of the deduced amino acids of *TaRAD51 *cDNA homoeologues with already reported *TaRAD51A1 *&*TaRAD51A2 *paralogues**. Conserved amino acids are indicated by black with a yellow background. The aa similarities between *TaRAD51A1 *and *TaRAD51*-7D is indicated by black with green background and aa similarities between *TaRAD51A2 *and *TaRAD51*-7A is indicated by black with grey background. Deletions are shown by *dashes* Click here for file

Additional file 2 **Accession numbers of the nucleotide sequences used in phylogenetic analysis**. Click here for file
