# Supplementary material for: The RAD51 and DMC1 homoeologous genes of bread wheat: cloning, molecular characterization and expression analysis
Source: BMC Res Notes. 2010 Sep 29;3:245. doi: 10.1186/1756-0500-3-245 (PMC2962619; doi:10.1186/1756-0500-3-245)
Supplement: Additional file 1 — Alignment and comparison of the deduced amino acids of TaRAD51 cDNA homoeologues with already reported TaRAD51A1 &TaRAD51A2 paralogues. Conserved amino acids are indicated by black with a yellow background. The aa similarities between TaRAD51A1 and TaRAD51-7D is indicated by black with green background and aa similarities between TaRAD51A2 and TaRAD51-7A is indicated by black with grey background. Deletions are shown by dashes [file 1756-0500-3-245-S1.PDF]

```
TaRAD51A1   MSSSAAHQKAAAAAPV-EEEAGEHGPFPIEQLQASGIAAVDVKKLKDAGLCTVESVAYSP 59
TaRAD51-7D  MSSSAAHQKAAAAAPV-EEEAGEHGPFPIEQLQASGIAAVDVKKLKDAGLCTVESVAYSP 59
TaRAD51-7B  MSSSAAHQKAAAAAPVEEEEAGEHGPFPIEHLQASGIAAVDVKKLKDAGLCTVESVAYSP 60
TaRAD51-7A  MSSAAAHQKAAAAAPVEEEEAGEHGPFPIEHLQASGIAAVDVKKLKDAGLCTVESVAYSP 60
TaRAD51A2   MSSAAAHQKAAAAAPVEEEEAGEHGPFPIEHLQASGIAAVDVKKLKDAGLCTVESVAYSP 60
            ***:************ *************:*****************************

TaRAD51A1   RKDLLQIKGISEAKVDKIIEAASKLVPLGFTSATQLHAQRLEIIQVTTGSRELDKILEGG 119
TaRAD51-7D  RKDLLQIKGISEAKVDKIIEAASKLVPLGFTSATQLHAQRLEIIQVTTGSRELDKILEGG 119
TaRAD51-7B  RKDLLQIKGISEAKVDKIIEAASKLVPLGFTSATQLHAQRLEIIQVTTGSRELDRILEGG 120
TaRAD51-7A  RKDLLQIKGISEAKVDKIIEAASKLVPLGFTSATQLHAQRLEIIQVTTGSRELDKILEGG 120
TaRAD51A2   RKDLLQIKGISEAKVDKIIEAASKLVPLGFTSATQLHAQRLEIIQVTTGSRELDKILEGG 120
            ******************************************************:*****

TaRAD51A1   IETGSITELYGEFRSGKTQLCHTLCVTCQLPLDQGGGEGKALYIDAEGTFRPQRLLQIAD 179
TaRAD51-7D  IETGSITELYGEFRSGKTQLCHTLCVTCQLPLDQGGGEGKALYIDAEGTFRPQRLLQIAD 179
TaRAD51-7B  IETGSITELYGEFRSGKTQLCHTLCVTCQLPLDQGGGEGKALYIDAEGTFRPQRLLQIAD 180
TaRAD51-7A  IETGSITELYGEFRSGKTQLCHTLCVTCQLPLDQGGGEGKALYIDAEGTFRPQRLLQIAD 180
TaRAD51A2   IETGSITELYGEFRSGKTQLCHTLCVTCQLPLDQGGGEGKALYIDAEGTFRPQRLLQIAD 180
            ************************************************************

TaRAD51A1   RFGLNGADVLENVAYARAYNTDHQSRLLLEAASMMVETRFALMVIDSATALYRTDFSGRG 239
TaRAD51-7D  RFGLNGADVLENVAYARAYNTDHQSRLLLEAASMMVETRFALMVIDSATALYRTDFSGRG 239
TaRAD51-7B  RFGLNGADVLENVAYARAYNTDHQSRLLLEAASMMVETRFALMVIDSATALYRTDFSGRG 240
TaRAD51-7A  RFGLNGADVLENVAYARAYNTDHQSRLLLEAASMMVETRFALMVIDSATALYRTDFSGRG 240
TaRAD51A2   RFGLYGADVLENVAYARAYNTDHQSRLLLEAASMMVETRFALMVIDSATALYRTDFSGRG 240
            **** *******************************************************

TaRAD51A1   ELSARQMHLAKFLRSLQKLADEFGVAVVISNQVVAQVDGGAMFAGPQIKPIGGNIMAHAS 299
TaRAD51-7D  ELSARQMHLAKFLRSLQKLADEFGVAVVISNQVVAQVDGGAMFAGPQIKPIGGNIMAHAS 299
TaRAD51-7B  ELSARQMHLAKFLRSLQKLADEFGVAVVISNQVVAQVDGGAMFAGPQIKPIGGNIMAHAS 300
TaRAD51-7A  ELSARQMHLAKFLRSLQKLADEFGVAVVISNQVVAQVDGGAMFAGPQIKPIGGNIMAHAS 300
TaRAD51A2   ELSARQMHLAKFLRSLQKLADEFGVAVVISNQVVAQVDGGAMFAGPQIKPIGGNIMAHAS 300
            ************************************************************

TaRAD51A1   TTRLYLRKGRAEERICKVVSSPCLAEAEARFQISPEGVTDVKD 342
TaRAD51-7D  TTRLYLRKGRAEERICKVVSSPCLAEAEARFQISPEGVTDVKD 342
TaRAD51-7B  TTRLYLRKGRAEERICKVVSSPCLAEAEARFQISPEGVTDVKD 343
TaRAD51-7A  TTRLYLRKGRAEERICKVVSSPCLAEAEARFQISPEGVTDVKD 343
TaRAD51A2   TTRLYLRKGKGGGADL
            *********
```
